# Supplementary material for: Process evaluation for the STAMINA randomised controlled trial: A protocol
Source: PLoS One. 2025 Jul 14;20(7):e0323275. doi: 10.1371/journal.pone.0323275 (PMC12258564; doi:10.1371/journal.pone.0323275)
Supplement: S1 File — (DOCX) [file pone.0323275.s001.docx]

**ADT Symptom Index**

We would like to know how much you have been affected by the hormone treatment you are receiving for your prostate cancer (also known as ADT).

| **Please circle a number below for each statement on the left** | | | | | | | |
| --- | --- | --- | --- | --- | --- | --- | --- |
| **Thinking about the past seven days** | | | **Not at all** | **A little bit** | **Some- what** | **Quite a bit** | **Very much** |
| 1 |  | I have been bothered by hot flushes / sudden sweats | **0** | **1** | **2** | **3** | **4** |
| 2 |  | I have felt tired | **0** | **1** | **2** | **3** | **4** |
| 3 |  | I have been concerned about putting on weight | **0** | **1** | **2** | **3** | **4** |
| 4 |  | I have been concerned about changes to the size of my chest/ breast area. | **0** | **1** | **2** | **3** | **4** |
| 5 |  | I have felt I have lost muscle strength | **0** | **1** | **2** | **3** | **4** |
| 6 |  | I have had difficulty concentrating on things | **0** | **1** | **2** | **3** | **4** |
| 7 |  | I have had difficulty remembering everyday things (e.g. words, where I have put something) | **0** | **1** | **2** | **3** | **4** |
| 8 |  | I have had difficulties sleeping | **0** | **1** | **2** | **3** | **4** |
| 9 |  | I have been concerned about increases in my waist size | **0** | **1** | **2** | **3** | **4** |
| 10 |  | I have experienced discomfort/ sensitivity in my nipples | **0** | **1** | **2** | **3** | **4** |
| 11 |  | I have been concerned about shrinkage of my penis/ genitals | **0** | **1** | **2** | **3** | **4** |
| 12 |  | I have had mood swings / felt emotional | **0** | **1** | **2** | **3** | **4** |
| 13 |  | I have been worried that my cancer will get worse / return | **0** | **1** | **2** | **3** | **4** |
| 14 |  | I have been bothered by joint or muscle aching/ stiffness | **0** | **1** | **2** | **3** | **4** |
| **Regardless of your current level of sexual activity, please answer the following questions. If you prefer**  **not to answer them, please tick this box. ☐** | | | **Not at all** | **A little bit** | **Some- what** | **Quite a bit** | **Very much** |
| 15 |  | My lack of sex drive (libido) has bothered me | **0** | **1** | **2** | **3** | **4** |
| 16 |  | My ability to get / maintain an erection has bothered me | **0** | **1** | **2** | **3** | **4** |
