# Supplementary material for: Process evaluation for the STAMINA randomised controlled trial: A protocol
Source: PLoS One. 2025 Jul 14;20(7):e0323275. doi: 10.1371/journal.pone.0323275 (PMC12258564; doi:10.1371/journal.pone.0323275)
Supplement: S2 — (DOCX) [file pone.0323275.s002.docx]

## Appendix 1 – Process Evaluation Timelines – Mapped onto the main trial timescales.

Fig 2: Data collection timelines

## Appendix 2 - Exercise Professional Theoretical domains framework questionnaire

Please enter today’s date: ........ / ........ / ................ (Day/Month/Year)

1. I am aware of the side effects of androgen deprivation therapy (ADT) for men with prostate cancer

| 1 | 2 | 3 | 4 | 5 |
| --- | --- | --- | --- | --- |
| Strongly disagree | Somewhat disagree | Neither agree or disagree | Somewhat agree | Strongly agree |

2. I am aware of the procedures for reporting adverse events and serious adverse events in the STAMINA programme

| 1 | 2 | 3 | 4 | 5 |
| --- | --- | --- | --- | --- |
| Strongly disagree | Somewhat disagree | Neither agree or disagree | Somewhat agree | Strongly agree |

3. I am aware of techniques to help support men on ADT for prostate cancer to change their behaviour

| 1 | 2 | 3 | 4 | 5 |
| --- | --- | --- | --- | --- |
| Strongly disagree | Somewhat disagree | Neither agree or disagree | Somewhat agree | Strongly agree |

4. I have the skills to deliver supervised exercise to men on ADT for prostate cancer

| 1 | 2 | 3 | 4 | 5 |
| --- | --- | --- | --- | --- |
| Strongly disagree | Somewhat disagree | Neither agree or disagree | Somewhat agree | Strongly agree |

5. I have the skills to communicate with the patient's NHS clinical team

| 1 | 2 | 3 | 4 | 5 |
| --- | --- | --- | --- | --- |
| Strongly disagree | Somewhat disagree | Neither agree or disagree | Somewhat agree | Strongly agree |

6. I have the skills to conduct and record results of a submaximal exercise test

| 1 | 2 | 3 | 4 | 5 |
| --- | --- | --- | --- | --- |
| Strongly disagree | Somewhat disagree | Neither agree or disagree | Somewhat agree | Strongly agree |

7. I can identify the difference between an adverse event and serious adverse event in line with research protocol

| 1 | 2 | 3 | 4 | 5 |
| --- | --- | --- | --- | --- |
| Strongly disagree | Somewhat disagree | Neither agree or disagree | Somewhat agree | Strongly agree |

8. I know how to tell when I'm not delivering exercise support as well as I could

| 1 | 2 | 3 | 4 | 5 |
| --- | --- | --- | --- | --- |
| Strongly disagree | Somewhat disagree | Neither agree or disagree | Somewhat agree | Strongly agree |

9. As an exercise professional, it is part of my role to provide behavioural support to men on ADT for prostate cancer.

| 1 | 2 | 3 | 4 | 5 |
| --- | --- | --- | --- | --- |
| Strongly disagree | Somewhat disagree | Neither agree or disagree | Somewhat agree | Strongly agree |

10. As an exercise professional, it is part of my role to collect data for research studies.

| 1 | 2 | 3 | 4 | 5 |
| --- | --- | --- | --- | --- |
| Strongly disagree | Somewhat disagree | Neither agree or disagree | Somewhat agree | Strongly agree |

11. I feel confident that I can deliver tailored exercise programmes to men on ADT for prostate cancer

| 1 | 2 | 3 | 4 | 5 |
| --- | --- | --- | --- | --- |
| Strongly disagree | Somewhat disagree | Neither agree or disagree | Somewhat agree | Strongly agree |

12. I feel confident that I can use techniques to motivate men on ADT to exercise at Nuffield Health

| 1 | 2 | 3 | 4 | 5 |
| --- | --- | --- | --- | --- |
| Strongly disagree | Somewhat disagree | Neither agree or disagree | Somewhat agree | Strongly agree |

13. I believe men on ADT for prostate cancer are capable of exercising twice a week

| 1 | 2 | 3 | 4 | 5 |
| --- | --- | --- | --- | --- |
| Strongly disagree | Somewhat disagree | Neither agree or disagree | Somewhat agree | Strongly agree |

14. I feel positive that men on ADT for prostate cancer will benefit from exercise

| 1 | 2 | 3 | 4 | 5 |
| --- | --- | --- | --- | --- |
| Strongly disagree | Somewhat disagree | Neither agree or disagree | Somewhat agree | Strongly agree |

15. Working with men on ADT for prostate cancer will help me achieve my professional goals

| 1 | 2 | 3 | 4 | 5 |
| --- | --- | --- | --- | --- |
| Strongly disagree | Somewhat disagree | Neither agree or disagree | Somewhat agree | Strongly agree |

16. I intend to support men on ADT for prostate cancer to exercise, if given the opportunity

| 1 | 2 | 3 | 4 | 5 |
| --- | --- | --- | --- | --- |
| Strongly disagree | Somewhat disagree | Neither agree or disagree | Somewhat agree | Strongly agree |

17. Men on ADT for prostate cancer will exercise twice a week at Nuffield Health

| 1 | 2 | 3 | 4 | 5 |
| --- | --- | --- | --- | --- |
| Strongly disagree | Somewhat disagree | Neither agree or disagree | Somewhat agree | Strongly agree |

18. Communicating with a patient's NHS clinical team will be challenging

| 1 | 2 | 3 | 4 | 5 |
| --- | --- | --- | --- | --- |
| Strongly disagree | Somewhat disagree | Neither agree or disagree | Somewhat agree | Strongly agree |

19. It will be rewarding for me to support men on ADT for prostate cancer to exercise

| 1 | 2 | 3 | 4 | 5 |
| --- | --- | --- | --- | --- |
| Strongly disagree | Somewhat disagree | Neither agree or disagree | Somewhat agree | Strongly agree |

20. I feel worried about delivering exercise to men on ADT for prostate cancer

| 1 | 2 | 3 | 4 | 5 |
| --- | --- | --- | --- | --- |
| Strongly disagree | Somewhat disagree | Neither agree or disagree | Somewhat agree | Strongly agree |

21. I feel worried about knowing what to do if things go wrong

| 1 | 2 | 3 | 4 | 5 |
| --- | --- | --- | --- | --- |
| Strongly disagree | Somewhat disagree | Neither agree or disagree | Somewhat agree | Strongly agree |

22. My colleagues support me working with men on ADT for prostate cancer

| 1 | 2 | 3 | 4 | 5 |
| --- | --- | --- | --- | --- |
| Strongly disagree | Somewhat disagree | Neither agree or disagree | Somewhat agree | Strongly agree |

23. I have the time and resources to support men on ADT for prostate cancer to exercise

| 1 | 2 | 3 | 4 | 5 |
| --- | --- | --- | --- | --- |
| Strongly disagree | Somewhat disagree | Neither agree or disagree | Somewhat agree | Strongly agree |

24. I have access to a secure email system to communicate patient data

| 1 | 2 | 3 | 4 | 5 |
| --- | --- | --- | --- | --- |
| Strongly disagree | Somewhat disagree | Neither agree or disagree | Somewhat agree | Strongly agree |

25. Nuffield Health have the resources to support me emotionally

| 1 | 2 | 3 | 4 | 5 |
| --- | --- | --- | --- | --- |
| Strongly disagree | Somewhat disagree | Neither agree or disagree | Somewhat agree | Strongly agree |

## Appendix 3 - Healthcare Professional Theoretical domains framework questionnaire

Please enter today’s date: ........ / ........ / ................ (Day/Month/Year)

1. I am aware of the NICE NG131 1.4.19 recommendations for exercise for men with prostate cancer on ADT.

| 1 | 2 | 3 | 4 | 5 |
| --- | --- | --- | --- | --- |
| Strongly disagree | Somewhat disagree | Neither agree or disagree | Somewhat agree | Strongly agree |

2. I am aware of the benefits of exercise for men with prostate cancer on ADT.

| 1 | 2 | 3 | 4 | 5 |
| --- | --- | --- | --- | --- |
| Strongly disagree | Somewhat disagree | Neither agree or disagree | Somewhat agree | Strongly agree |

3. I have the skills to deliver exercise support and make an exercise referral in line with NICE NG131 1.4.19 recommendations to men with prostate cancer on ADT.

| 1 | 2 | 3 | 4 | 5 |
| --- | --- | --- | --- | --- |
| Strongly disagree | Somewhat disagree | Neither agree or disagree | Somewhat agree | Strongly agree |

4. I always remember to discuss exercise with men with prostate cancer on ADT during consultations

| 1 | 2 | 3 | 4 | 5 |
| --- | --- | --- | --- | --- |
| Strongly disagree | Somewhat disagree | Neither agree or disagree | Somewhat agree | Strongly agree |

5. I have made a plan about how to deliver and check, that I offer exercise support and referral, to all men with prostate cancer on ADT.

| 1 | 2 | 3 | 4 | 5 |
| --- | --- | --- | --- | --- |
| Strongly disagree | Somewhat disagree | Neither agree or disagree | Somewhat agree | Strongly agree |

6. I see it as part of my professional role to discuss and support exercise for my patients with prostate cancer on ADT.

| 1 | 2 | 3 | 4 | 5 |
| --- | --- | --- | --- | --- |
| Strongly disagree | Somewhat disagree | Neither agree or disagree | Somewhat agree | Strongly agree |

7. I feel confident that I can assess the suitability of men with prostate cancer on ADT to exercise.

| 1 | 2 | 3 | 4 | 5 |
| --- | --- | --- | --- | --- |
| Strongly disagree | Somewhat disagree | Neither agree or disagree | Somewhat agree | Strongly agree |

8. I feel confident that all men with prostate cancer on ADT will receive exercise support and referral in line with NICE NG131 1.4.19 recommendations.

| 1 | 2 | 3 | 4 | 5 |
| --- | --- | --- | --- | --- |
| Strongly disagree | Somewhat disagree | Neither agree or disagree | Somewhat agree | Strongly agree |

9. Exercise in line with the NICE NG131 1.4.19 recommendations will be beneficial for men with prostate cancer on ADT.

| 1 | 2 | 3 | 4 | 5 |
| --- | --- | --- | --- | --- |
| Strongly disagree | Somewhat disagree | Neither agree or disagree | Somewhat agree | Strongly agree |

10. Men with advanced prostate cancer on ADT will be capable of taking part in exercise in line with the NICE NG131 1.4.19 recommendations.

| 1 | 2 | 3 | 4 | 5 |
| --- | --- | --- | --- | --- |
| Strongly disagree | Somewhat disagree | Neither agree or disagree | Somewhat agree | Strongly agree |

11. I intend to discuss exercise with all my patients who have prostate cancer on ADT when I see them in clinic.

| 1 | 2 | 3 | 4 | 5 |
| --- | --- | --- | --- | --- |
| Strongly disagree | Somewhat disagree | Neither agree or disagree | Somewhat agree | Strongly agree |

12. I want to deliver exercise support as much as I want to deliver the other aspects of my role/tasks that I need to deliver to men with prostate cancer on ADT.

| 1 | 2 | 3 | 4 | 5 |
| --- | --- | --- | --- | --- |
| Strongly disagree | Somewhat disagree | Neither agree or disagree | Somewhat agree | Strongly agree |

13. There will be positive benefits for me as a healthcare professional, if I deliver exercise support and referral in line with recommendations to all men I see in clinic with prostate cancer on ADT.

| 1 | 2 | 3 | 4 | 5 |
| --- | --- | --- | --- | --- |
| Strongly disagree | Somewhat disagree | Neither agree or disagree | Somewhat agree | Strongly agree |

14. I feel positive about delivering exercise support and referral in line with recommendations to men with prostate cancer on ADT.

| 1 | 2 | 3 | 4 | 5 |
| --- | --- | --- | --- | --- |
| Strongly disagree | Somewhat disagree | Neither agree or disagree | Somewhat agree | Strongly agree |

15. I have enough time and resources to be able to discuss exercise with all my patients with prostate cancer on ADT during all consultations

| 1 | 2 | 3 | 4 | 5 |
| --- | --- | --- | --- | --- |
| Strongly disagree | Somewhat disagree | Neither agree or disagree | Somewhat agree | Strongly agree |

16. Fellow healthcare professionals expect that I should be discussing exercise in all consultations with men with prostate cancer on ADT.

| 1 | 2 | 3 | 4 | 5 |
| --- | --- | --- | --- | --- |
| Strongly disagree | Somewhat disagree | Neither agree or disagree | Somewhat agree | Strongly agree |

17. Men with prostate cancer on ADT expect that I should be discussing exercise in all consultations with men with prostate cancer on ADT.

| 1 | 2 | 3 | 4 | 5 |
| --- | --- | --- | --- | --- |
| Strongly disagree | Somewhat disagree | Neither agree or disagree | Somewhat agree | Strongly agree |

## Appendix 4 - Exercise Professional Theoretical domains framework questionnaire: remote

Please enter today’s date: ........ / ........ / ................ (Day/Month/Year)

1. I am aware how to monitor exercise intensity ***remotely***

| 1 | 2 | 3 | 4 | 5 |
| --- | --- | --- | --- | --- |
| Strongly disagree | Somewhat disagree | Neither agree or disagree | Somewhat agree | Strongly agree |

2. I am aware of techniques to help support men with prostate cancer to exercise ***remotely***

| 1 | 2 | 3 | 4 | 5 |
| --- | --- | --- | --- | --- |
| Strongly disagree | Somewhat disagree | Neither agree or disagree | Somewhat agree | Strongly agree |

3. I have the skills to supervise exercise ***remotely*** for a man with prostate cancer on ADT

| 1 | 2 | 3 | 4 | 5 |
| --- | --- | --- | --- | --- |
| Strongly disagree | Somewhat disagree | Neither agree or disagree | Somewhat agree | Strongly agree |

4. I have the skills to supervise exercise ***remotely*** to a ***small group*** of men with prostate cancer on ADT (up to 5 men)

| 1 | 2 | 3 | 4 | 5 |
| --- | --- | --- | --- | --- |
| Strongly disagree | Somewhat disagree | Neither agree or disagree | Somewhat agree | Strongly agree |

5. I have the skills to provide tailored behavioural support ***remotely***

| 1 | 2 | 3 | 4 | 5 |
| --- | --- | --- | --- | --- |
| Strongly disagree | Somewhat disagree | Neither agree or disagree | Somewhat agree | Strongly agree |

6. I can remember how to adapt exercise for men on ADT

| 1 | 2 | 3 | 4 | 5 |
| --- | --- | --- | --- | --- |
| Strongly disagree | Somewhat disagree | Neither agree or disagree | Somewhat agree | Strongly agree |

7. I know how to tell when I'm not delivering exercise support as well as I could

| 1 | 2 | 3 | 4 | 5 |
| --- | --- | --- | --- | --- |
| Strongly disagree | Somewhat disagree | Neither agree or disagree | Somewhat agree | Strongly agree |

8. As an exercise professional, it is part of my role to deliver supervised exercise ***remotely***

| 1 | 2 | 3 | 4 | 5 |
| --- | --- | --- | --- | --- |
| Strongly disagree | Somewhat disagree | Neither agree or disagree | Somewhat agree | Strongly agree |

9. I feel confident that I can ***create*** a tailored exercise programme suitable for the home environment

| 1 | 2 | 3 | 4 | 5 |
| --- | --- | --- | --- | --- |
| Strongly disagree | Somewhat disagree | Neither agree or disagree | Somewhat agree | Strongly agree |

10. I feel confident that I can use techniques to motivate patients to exercise at home

| 1 | 2 | 3 | 4 | 5 |
| --- | --- | --- | --- | --- |
| Strongly disagree | Somewhat disagree | Neither agree or disagree | Somewhat agree | Strongly agree |

11. I feel confident that I can deliver exercise remotely using teleconferencing software e.g. Microsoft Teams

| 1 | 2 | 3 | 4 | 5 |
| --- | --- | --- | --- | --- |
| Strongly disagree | Somewhat disagree | Neither agree or disagree | Somewhat agree | Strongly agree |

12. I feel positive that men with prostate cancer will benefit from a supervised home-based exercise programme

| 1 | 2 | 3 | 4 | 5 |
| --- | --- | --- | --- | --- |
| Strongly disagree | Somewhat disagree | Neither agree or disagree | Somewhat agree | Strongly agree |

13. I feel positive that men with prostate cancer will be happy to exercise remotely using technology

| 1 | 2 | 3 | 4 | 5 |
| --- | --- | --- | --- | --- |
| Strongly disagree | Somewhat disagree | Neither agree or disagree | Somewhat agree | Strongly agree |

14. I believe I can develop a trusting relationship with patients remotely

| 1 | 2 | 3 | 4 | 5 |
| --- | --- | --- | --- | --- |
| Strongly disagree | Somewhat disagree | Neither agree or disagree | Somewhat agree | Strongly agree |

15. I believe I can review patient progress remotely

| 1 | 2 | 3 | 4 | 5 |
| --- | --- | --- | --- | --- |
| Strongly disagree | Somewhat disagree | Neither agree or disagree | Somewhat agree | Strongly agree |

16. I intend to deliver remote supervision to men with prostate cancer, if given the opportunity

| 1 | 2 | 3 | 4 | 5 |
| --- | --- | --- | --- | --- |
| Strongly disagree | Somewhat disagree | Neither agree or disagree | Somewhat agree | Strongly agree |

17. Delivering supervised exercise remotely will help me achieve my professional goals

| 1 | 2 | 3 | 4 | 5 |
| --- | --- | --- | --- | --- |
| Strongly disagree | Somewhat disagree | Neither agree or disagree | Somewhat agree | Strongly agree |

18. It will be rewarding for me to work men with prostate cancer on ADT

| 1 | 2 | 3 | 4 | 5 |
| --- | --- | --- | --- | --- |
| Strongly disagree | Somewhat disagree | Neither agree or disagree | Somewhat agree | Strongly agree |

19. I feel worried about supervising exercise remotely

| 1 | 2 | 3 | 4 | 5 |
| --- | --- | --- | --- | --- |
| Strongly disagree | Somewhat disagree | Neither agree or disagree | Somewhat agree | Strongly agree |

20. I feel worried about knowing what to do if things go wrong

| 1 | 2 | 3 | 4 | 5 |
| --- | --- | --- | --- | --- |
| Strongly disagree | Somewhat disagree | Neither agree or disagree | Somewhat agree | Strongly agree |

21. I have the resources to deliver exercise via video conferencing software, e.g. Microsoft Teams

| 1 | 2 | 3 | 4 | 5 |
| --- | --- | --- | --- | --- |
| Strongly disagree | Somewhat disagree | Neither agree or disagree | Somewhat agree | Strongly agree |

22. I have the resources to monitor exercise intensity remotely

| 1 | 2 | 3 | 4 | 5 |
| --- | --- | --- | --- | --- |
| Strongly disagree | Somewhat disagree | Neither agree or disagree | Somewhat agree | Strongly agree |

23. My Nuffield colleagues support me working with men with prostate cancer

| 1 | 2 | 3 | 4 | 5 |
| --- | --- | --- | --- | --- |
| Strongly disagree | Somewhat disagree | Neither agree or disagree | Somewhat agree | Strongly agree |

## Appendix 5 –Sampling framework for fidelity analysis of CES:participant interactions

- A minimum of 10 different exercise professionals

- A minimum of 40 different participants

- A minimum of 10 induction sessions

- A minimum of 10 week 12 review sessions

- A minimum of 2 month 6 review sessions

- A minimum of 2 month 12 review sessions
