# Supplementary material for: Process evaluation for the STAMINA randomised controlled trial: A protocol
Source: PLoS One. 2025 Jul 14;20(7):e0323275. doi: 10.1371/journal.pone.0323275 (PMC12258564; doi:10.1371/journal.pone.0323275)
Supplement: S3 — (DOCX) [file pone.0323275.s003.docx]

**Topic guide for interviews with men who have received the exercise intervention in work package** (Based on Sekhon 2017 and NPT)

- How did you find out about the STAMINA lifestyle programme?
  - Possible probes: Who spoke to you about the programme? What did they say? Face-to-face or remote? Have they recommended exercise in the past? Did they give you an information sheet? How helpful was this process?
- After that [the hospital had spoken to you about STAMINA] did someone call you to ask you if you were interested in taking part in the programme?
  - How did you find this process? How long was it after you had been to/ spoken about it at hospital? What did they say? How similar/ different was it to what you had heard at the hospital?
- You then had a longer conversation for the safety to exercise check.
  - How did you find this process?
- Were there any follow up calls after that call? (If yes, what was that for etc?)
- Have you called the team for any advice since then? (If yes, what was that for etc?)
- How would you describe your exercise behaviour before you started the STAMINA programme?
  - Possible probes: type, frequency, intensity of exercise.
  - How important do you think exercise is for people with prostate cancer? Probe: before STAMINA and now
  - How confident do you feel to exercise? Probe: before STAMINA and now.
- How did you find the STAMINA lifestyle programme?
  - Possible Probes: What did you like/didn’t you like?

Feelings about group supervision versus 1-2-1 versus unsupervised? Incl. personal tailoring of exercise

components within group classes

Confidence in group classes?

Influence of any other health issues on feelings about the programme?

Changes in feelings about the classes over time?

Any PCa treatment during the exercise programme? How did that work

with the exercise programme?

Feelings about HCP discussion before referral – what was discussed

Feelings about patient materials e.g. info book and training diary

Feelings about review sessions with CES

- What do you think you got out of the STAMINA programme ?
  - Possible Probes: Do you think the intervention has had an impact on your life? In what way?

Influence on other health issues, effects of ongoing treatment

Changes in feelings about the STAMINA lifestyle programme over time?

Changes in perceptions about exercise or dietary modification?

- Do you believe the STAMINA programme can be helpful to men like you?
  - Possible Probes: in what way, how, why?
- How has the programme had an affect on aspects of your life beyond exercise?
  - Probes: for example on what you eat, other lifestyle behaviours?

Impact on broader quality of life, social aspects etc

- What did participation in STAMINA involve for you?
  - Possible Probes: time, money, discomfort, inconvenience, worry, missing out on other things
- What did you feel about completing the personal exercise diary?/ how did it go?
- Did your progress in the exercise programme get fed back to your health care team (e.g at the hospital)?
  - Probes: how, how often, how did they respond, how much discussion?
  - Your feelings about feedback, was this helpful to you, was this helpful to your healthcare team

{for those who dropped out of the intervention}

- Can you say why you weren’t able to/ decided not to complete the STAMINA exercise programme?
  - Probes: are you continuing to exercise outside the programme? What are you doing? How often? Routines? Do you have future pans around exercise?

{for those who are still doing the intervention}

- How easy do you feel it will be to continue exercising when the programme has finished?

Possible probes: plans

{for those who have completed the intervention}

- How easy has it been to continue exercising?
  - Probe: what are they actually doing, plans, routines?

If participation in exercise has fallen off: why? What might have helped?

{for those who were allocated a heart rate monitor}

- How did you find using the heart rate monitor?
- What do you think you got out of using the heart rate monitor?
- Do you believe heart rate monitors can be helpful to men like you?
- What did using the heart rate monitor involve for you?
  - PROBE: putting it on, recording sessions, syncing data
- Is there anything else you’d like to say about the intervention?

**Topic guide for interviews with CESs**

- Can you describe your role and what was done to get STAMINA going at your site?
- What has helped to keep STAMINA going at your site?

• How did you find delivering the intervention?

o Experience of delivering the intervention? Probe: contacting patients, delivering inductions, 1-2-1 versus group, progress reviews, behavioural support, recording data.

o Feelings about the intervention? Delivering group supervision ? Individual tailoring within the group supervision. Probe: 1-2-1 versus group. Face-to-face versus remote.

• What did delivering the intervention involve?

o What did it involve in terms of time/ opportunity/ effort to train to, and then deliver, the intervention?

How well does STAMINA embed into your current ways of working at Nuffield Health?

- Who do you think is most suitable in your team to be delivering STAMINA?

• Do you believe this sort of intervention can be helpful to men in with prostate cancer?

o In what way? How?

• Do you feel the role of delivering this intervention is appropriate for exercise professionals such as yourself?

- - Do you have any concerns about this role?

• Do you have any concerns or worries about the intervention?

- Probe: Thoughts before training, after training.
- Did you discuss any other lifestyle changes with the men in addition to exercise?

What kind of things (e.g. how they eat etc how often did you have these sort of discussions?, what prompted these discussions? how were they incorporated into the STAMINA Lifestyle Intervention? what effect did they have on the men?

What did you think about the progress reports to the healthcare team?

Prompts: How useful? How necessary? Effort involved? How completed? Any input from the men themselves? Any feedback to you from the health care professionals? Any feedback regarding the progress report from the men?

Any improvements or changes you would make to the progress reports?

How did you find any other aspects of communication related to the STAMINA lifestyle intervention?

Prompts: with the research team? Related to any adverse events? Were you able to voice concerns about any aspects of delivering the intervention easily? Did you feel responses to any concerns were timely and appropriate? With HCPs?

{for those who provided heart rate monitors}

- How did you find supporting men to use the heart rate monitors?
- What do you think you got out of using the heart rate monitors?
- Do you believe heart rate monitors can be helpful for men with prostate cancer?
- What did providing heart rate monitors involve for you?
  - PROBE: putting it on, recording sessions, syncing data
- Can you describe any changes you would make to STAMINA ahead of a future roll out?

• Is there anything else you’d like to say about your experience of delivering the intervention or about the intervention in general?

**Topic guide for interviews with cancer clinicians caring for men who have received the STAMINA Lifestyle Intervention**

Can you describe your role and what was done to get STAMINA going at your site?

- What has helped to keep STAMINA going at your site?
- How did you find identifying eligible participants/ providing lifestyle support/ sending the referral/ maintaining the ADT log?

o Experience of delivering the intervention? (Probe: Challenges, barriers, what helped, patient response, preconceptions)

o Feelings about the intervention?

- - Do you feel the clinical team have a role in delivering the intervention?
  - Did any staff act as STAMINA “champions”? What role did the champions have? What did they do? What (if any) impact?
  - Who do you think is most suitable in your team to be delivering STAMINA?
- What did it involve in terms of time/ opportunity/ effort to train to, and then deliver, the intervention?

How well does STAMINA embed into your current pathway?

- Do you feel your clinical practice has changed as a result of being involved in the study?
- How? In what way? Examples? With other patients? The whole team?
- Were there any other impacts on your clinical practise?

• Do you believe this sort of intervention can be helpful to men in with prostate cancer?

o In what way?

- Did you notice any changes related to the intervention in your patients?
  - What? How do you think they came about? (Probe: OUC versus SLI)
  - Have these changes been sustained? Do you believe they can/will be sustained?
  - Any lifestyle changes beyond exercise?
- How did you find receiving the feedback reports from Nuffield Health?

Prompts: How useful? How necessary? Were they understandable? How easy for you to respond to them? Any feedback from the men themselves?

- How comfortable/ easy did you find talking about the STAMINA programme and their progress with patients?
  - Probe: how relevant to your clinical consultation?

How did you find any other aspects of communication related to the STAMINA lifestyle intervention?

Prompts: with the research team? Related to any adverse events? Were you able to voice concerns about any aspects the intervention easily? Did you feel responses to any concerns were timely and appropriate?

- Having referred patients to programme what are your general thoughts about it?
  - Prompts: purpose of the programme? Role of the programme? Value of the programme?
- Should the STAMINA intervention be rolled out across the NHS?
  - Prompts: How do you think that would it work in routine care?
  - Can you describe any changes you would make to STAMINA ahead of a future roll out?

**Topic Guide for GPs Commissioners and other stakeholders**

- How familiar are you with the STAMINA programme? {if not familiar give brief presentations/explanation}
- What are your general thoughts/ first impressions on hearing about the programme?
  - Prompts purpose of the programme? Role of the programme? Value of the programme?
- Should the STAMINA intervention be rolled out across the NHS?
  - Prompts: How do you think that would it work in routine care?
  - How best to approach getting buy in to adopt STAMINA across the NHS?
  - Explore their role and how they might influence implementation?
- What would it take for the intervention to be rolled out across the NHS/ implemented into the standard NHS cancer care pathway?
  - Level of evidence/ benefit
  - Resources, roles communication pathways?
- What might assist/promote the intervention to be adopted by the NHS?
- What might stop the intervention from being adopted by the NHS?
- How likely is it that such an intervention might be rolled out across the NHS?
- What is the appetite for implementing interventions such as these across the NHS?
- What outcomes should be routinely collected if such a programme were to be rolled out across the NHS?

• Is there anything else you’d like to say about your experience of delivering the intervention or about the intervention in general?

**Topic guide for interviews with men who have received Optimised Usual Care**

- How did you find out about the STAMINA lifestyle programme?
  - Possible probes: Who spoke to you about the programme? What did they say? Face-to-face or remote? Have they recommended exercise in the past? Did they give you an information sheet? How helpful was this process?
- After that [the hospital had spoken to you about STAMINA] did someone call you to ask you if you were interested in taking part in the programme?
  - How did you find this process? How long was it after you had been to/ spoken about it at hospital? What did they say? How similar/ different was it to what you had heard at the hospital?
- You then had a longer conversation for the safety to exercise check.
  - How did you find this process?
- Were there any follow up calls after that call? (If yes, what was that for etc?)
- Have you called the team for any advice since then? (If yes, what was that for etc?)
- How would you describe your exercise behaviour before you were involved in this research study?
  - Possible probes: type, frequency, intensity of exercise.
  - How important do you think exercise is for people with prostate cancer? Probe: before you heard about it and now
  - How confident do you feel to exercise? Probe: before STAMINA and now.

• How helpful was the information in the booklets you were sent?

- When you received the booklet was the information new to you or was it something you already knew about?
- Of the activities / groups listed were there any you had taken part in before / thought about taking part in before?
- Did you follow the advice to start exercising / exercise more?
  - When did you start?
  - What exercise have you taken up?
  - How did you find out about it?
  - Why did you choose that particular group / exercise?
  - Is it something you do alone, or in a group?
- How helpful did you find the advice from the health care professionals?
- Did you have any worries about taking part in exercise?
- Did the recommendation by the HCP influence your decision to take part?
- How would you describe your exercise behaviour now?
  - Have you carried on exercising? Do you think you will carry on exercising? What do you think will keep you going with your exercise?
  - Possible probes: type, frequency, intensity of exercise.
- What do you think you have got out of exercising?
  - Possible Probes: Do you think taking up exercise has had an impact on your life? In what way?

Influence on other health issues, effects of ongoing treatment

- - Changes in feelings about exercise over time?
- Do you believe exercise can be helpful to men like you?
  - Possible Probes: in what way, how, why?
- How has taking up exercise impacted other areas of your life
  - Probes: for example on what you eat, other lifestyle behaviours?

Impact on broader quality of life, social aspects etc

- What did up exercise / doing more exercise involve for you?
  - Possible Probes: time, money, discomfort, inconvenience, worry, missing out on other things
- Did you discuss your exercise regime with your health care team (e.g at the hospital)?
  - Probes: how, how often, how did they respond, how much discussion?
  - Your feelings about feedback/discussing your progress, was this helpful to you, was this helpful to your healthcare team? Thoughts on the patient booklets?

{for those who didn’t take up exercise}

- Can you say why you weren’t able to/ decided not to take up exercise as recommended by your HCP?
  - What do you think would have helped you to take up exercise?
  - Probes: any barriers? Do you have future plans around exercise?

{for those who are still exercising}

- How easy has it been to continue exercising?
  - Probe: what are they actually doing, plans, routines?

If participation in exercise has fallen off: why? What might have helped?

- Is there anything else you’d like to say?
