# Supplementary material for: Process evaluation for the STAMINA randomised controlled trial: A protocol
Source: PLoS One. 2025 Jul 14;20(7):e0323275. doi: 10.1371/journal.pone.0323275 (PMC12258564; doi:10.1371/journal.pone.0323275)
Supplement: Attachment — Submitted filename: Plos response.docx [file pone.0323275.s005.docx]

PE protocol peer reviews response

Journal Requirements:

When submitting your revision, we need you to address these additional requirements.

1. Please ensure that your manuscript meets PLOS ONE's style requirements, including those for file naming. The PLOS ONE style templates can be found at 
https://journals.plos.org/plosone/s/file?id=wjVg/PLOSOne_formatting_sample_main_body.pdf and 
https://journals.plos.org/plosone/s/file?id=ba62/PLOSOne_formatting_sample_title_authors_affiliations.pdf

Title page updated. File naming and figure naming updated.

2. Thank you for stating the following financial disclosure: 
This work is funded by the NIHR Programme Grants for Applied Research (PGfAR) RP-PG-1016-20007. The views expressed are those of the author(s) and not necessarily those of the NHS, the NIHR or the Department of Health and Social Care.

Please state what role the funders took in the study.  If the funders had no role, please state: ""The funders had no role in study design, data collection and analysis, decision to publish, or preparation of the manuscript.""

If this statement is not correct you must amend it as needed. 
Please include this amended Role of Funder statement in your cover letter; we will change the online submission form on your behalf.

Added to cover letter

3. Ethics statement only appears at the end of the manuscript:
Your ethics statement should only appear in the Methods section of your manuscript. If your ethics statement is written in any section besides the Methods, please move it to the Methods section and delete it from any other section. Please ensure that your ethics statement is included in your manuscript, as the ethics statement entered into the online submission form will not be published alongside your manuscript.

 Amended as requested - see lines 156-157

4. Please include a caption for figure 2.

 Apologies it seem the text box fell off the document border this has been moved upwards – see page 28 “Fig 2: Data collection timelines”

5. We note you have included a table to which you do not refer in the text of your manuscript. Please ensure that you refer to Table 5, 6, 7 and 9 in your text; if accepted, production will need this reference to link the reader to the Table.

 There are no tables 5-9, you may be detecting the copies of tables 1-4 at the end of the manuscript. These have now been removed.

6. Please include captions for your Supporting Information files at the end of your manuscript, and update any in-text citations to match accordingly. Please see our Supporting Information guidelines for more information: <http://journals.plos.org/plosone/s/supporting-information>.

Amended as requested – added to line 518

# My response to editors

1. Style and filenames
2. Funding statement
3. Ethics statement
4. Figure 2 captions
5. Refer to tables
6. Caption supporting information

[Note: HTML markup is below. Please do not edit.]

Reviewers' comments:

Reviewer's Responses to Questions

**Comments to the Author**

1. Does the manuscript provide a valid rationale for the proposed study, with clearly identified and justified research questions?

The research question outlined is expected to address a valid academic problem or topic and contribute to the base of knowledge in the field.

Reviewer #1: Yes

Reviewer #2: Yes

Reviewer #3: Yes

2. Is the protocol technically sound and planned in a manner that will lead to a meaningful outcome and allow testing the stated hypotheses?

The manuscript should describe the methods in sufficient detail to prevent undisclosed flexibility in the experimental procedure or analysis pipeline, including sufficient outcome-neutral conditions (e.g. necessary controls, absence of floor or ceiling effects) to test the proposed hypotheses and a statistical power analysis where applicable. As there may be aspects of the methodology and analysis which can only be refined once the work is undertaken, authors should outline potential assumptions and explicitly describe what aspects of the proposed analyses, if any, are exploratory.

Reviewer #1: Yes

Reviewer #2: Yes

Reviewer #3: Partly

3. Is the methodology feasible and described in sufficient detail to allow the work to be replicable?


Descriptions of methods and materials in the protocol should be reported in sufficient detail for another researcher to reproduce all experiments and analyses. The protocol should describe the appropriate controls, sample size calculations, and replication needed to ensure that the data are robust and reproducible.

Reviewer #1: Yes

Reviewer #2: Yes

Reviewer #3: Yes

4. Have the authors described where all data underlying the findings will be made available when the study is complete?

The [PLOS Data policy](https://journals.plos.org/plosone/s/materials-and-software-sharing) requires authors to make all data underlying the findings described in their manuscript fully available without restriction, with rare exception, at the time of publication. The data should be provided as part of the manuscript or its supporting information, or deposited to a public repository. For example, in addition to summary statistics, the data points behind means, medians and variance measures should be available. If there are restrictions on publicly sharing data—e.g. participant privacy or use of data from a third party—those must be specified.

Reviewer #1: Yes

Reviewer #2: No

Reviewer #3: Yes

5. Is the manuscript presented in an intelligible fashion and written in standard English?

PLOS ONE does not copyedit accepted manuscripts, so the language in submitted articles must be clear, correct, and unambiguous. Any typographical or grammatical errors should be corrected at revision, so please note any specific errors here.

Reviewer #1: Yes

Reviewer #2: Yes

Reviewer #3: Yes

6. Review Comments to the Author

Please use the space provided to explain your answers to the questions above and, if applicable, provide comments about issues authors must address before this protocol can be accepted for publication. You may also include additional comments for the author, including concerns about research or publication ethics.

You may also provide optional suggestions and comments to authors that they might find helpful in planning their study.


(Please upload your review as an attachment if it exceeds 20,000 characters)

# My response to reviewers

Reviewer #1:

1.Can the authors clarify the rationale behind the chosen mixed-methods process evaluation design and its alignment with the study's objectives?

The mixed-methods design was chosen in line with the latest guidance concerning the evaluation of complex interventions and process evaluations. This led us to use the UK Medical Research Council and Linnan and Steckler evaluation frameworks as stated on lines 172-173. It would not be possible to address the various aims of this process evaluation without employing a combination of qualitative and quantitative methods as some aims are incompatible with a particular type of methods. Qualitative methods are often required for questions of understanding. Quantitative methods are well suited to descriptive questions which, given the scale of this evaluation and inevitable resource constraints, offer efficient means of collecting data.

Furthermore, in the seminal paper by Oakley et al 2006 in The BMJ it is clearly articulated that in process evaluations of randomised controlled trials should be informed by both quantitative and qualitative data - <https://pmc.ncbi.nlm.nih.gov/articles/PMC1370978/>

2. How do the authors ensure the consistency (fidelity) of training and intervention delivery across different sites, especially since the trial involves multiple healthcare professionals and exercise professionals?

The STAMINA trial is a pragmatic study whereby healthcare professionals and exercise professionals (i.e. non-research staff) will deliver the interventions in a real-world situation. As such the potential for variation in the delivery of training and the intervention is to be anticipated. Fidelity is conceptualised in this study according the National Institute of Health Behavioural Change Consortium framework (line 25, line 286 – reference 28) and we sought to preserve fidelity according to the guidance in this framework.

With regards to the fidelity of training – in cases of both healthcare professional training and exercise professional training standardised training packages were used by an intentionally small number of training facilitators using a purpose-designed training manual. In the case of the exercise professional training, all training was delivered by one facilitator. This was not guaranteed by design but will be demonstrated in the published results (see facilitator characteristics in Table 1).

With regards to fidelity of delivery – Healthcare professionals received physical prompts to take to clinic visits to facilitate delivery of the target behaviours in practice. Exercise professionals received a detailed intervention delivery manual with step-by-step instructions regarding the target behaviours. Further details of these are intended to be published in other works.

3. The study proposes integrating findings from different methods (e.g., interviews, process outcomes). Can the authors provide more specific details about how they plan to triangulate these findings to draw meaningful conclusions?

There are several ways by which the design of this evaluation gives us the opportunity to integrate findings and triangulate data from different sources. Firstly, from Table 1 it is clear that there are many instances where data from multiple sources are integrated to provide a more comprehensive insight into the behaviour of individual process evaluation component. Next, the relationship between these components and how they are presumed to act in a causal chain of events that produce the intervention outcomes is described in the programme logic (Fig 1). From this we have a theoretical basis upon which to consider the behaviour of individual components was influenced by others. It is less clear *a priori* exactly how this will emerge because there are many possible ways that the process data could deviate from a perfectly homogenous prespecified model.

As a simplified single example of how data might be integrated, we may observe that there are high rates of dropout from the intervention that vary by site. Interview data from participants healthcare professionals and/or exercise professionals might elucidate some factors exist may have negative influences on the acceptability of the intervention.

It is probably not worthwhile to try to anticipate all examples of how we might integrate findings because there are too many possibilities. To put it another way there is a both a large potential problem space and solution space meaning that attempting to concisely define all possible problems and potential solutions is impossible.

For the reasons explained above, there are very, very few examples of how process evaluation protocols specify how their results would be integrated. Here is the best example we can find of how this a process evaluation protocol would address your question – clearly there are limitations to the level of detail that can be provided in a such a protocol:

“Integration of the qualitative and quantitative case study data regarding implementation of different components of the intervention will allow detailed evaluation of the quality of intervention implementation in each case study site. For example, quantitative data relating to the number and type of patient problems identified for the agenda and included in the health plan can be integrated with observational data about how clinicians addressed agenda setting and health planning. The additional integration of interview data about patients’ and clinicians’ experiences of reviews will provide in-depth insight into those processes.”

Mann C, Shaw A, Guthrie B, et al. Protocol for a process evaluation of a cluster randomised controlled trial to improve management of multimorbidity in general practice: the 3D study. BMJ Open 2016;6:e011260. doi:10.1136/bmjopen-2016-011260

4. The authors mention some limitations of their approach, particularly regarding fidelity and the absence of audio recordings of healthcare professional-patient interactions. Could the authors elaborate on alternative strategies to mitigate the effects of these limitations?

There is limited evidence-based mitigation possible for mitigated the limitations of this particular method of data collection. We can’t know confidently what the effect the HCPs had on patient behaviours, but we could be explore this in future implementation work if the trial has a positive outcome. Some information regarding professional-patient interactions may be picked up in patient interviews – we explicitly asked patients in interviews about their experiences with HCPs and HCP target behaviours.

Reviewer #2:

Well designed evaluation study protocol and well written manuscript. Congratulations. Few comments/suggestions are attached
Page 3-Line no-53-56: just wondering whether the listed adverse events would be evaluated pre and post intervention

Thank you for your kind comment. Only some of these adverse consequences of ADT are evaluated as part of the trial including fatigue, adiposity indices, body mass, sexual dysfunction. Some of these adverse consequences are not suitable to study over only the 12-month follow-up period of this study. Note that the measurement of these outcomes is addressed by the trial protocol and not this process evaluation.

Page 7 Line no. 150 and 19 and line number -Implementation team doing the process evaluation-Need to incorporate suggestion to strengthen the manuscript-Are these going to be evaluated pre and post intervention?

Though it is unclear what is being asked here, please refer to Fig 2 to see the timelines for evaluation of the process evaluation components in relation to intervention delivery.
Page.7: table numbering to be corrected for column as currently number continues from 1 to 59. Each column and row should start with 1

The numbering in Table 1 deliberately continues from 1-59 to facilitate cross referencing of these data sources in Table 2. An alternative numbering of A1, B2, etc could be used instead.
Page 11 -Line no.163: Omission to be rectified-Add ADT

Thank you for spotting this formatting error, it has now been rectified.
Page 15 Table 4: Suggestion for inclusion--Facilitating factors that helped/induced sustenance of the participants in the programme could be added

We appreciate the suggestion to include this factor, but we would like to remain consistent within the externally defined theoretical framework that we are applying – see reference 26 below. In any case topic guides for the interviews have already been approved as part of the ethics application and it may not be timely to request and amendment. The framework was semi-structured so elements related to sustenance might have been captured and can discussed in the study results if appropriate.

Sekhon M, Cartwright M, Francis JJ. Acceptability of healthcare interventions: an overview of reviews and development of a theoretical framework. BMC Health Serv Res. 2017 Jan 26;17(1):88.

Page 15 Table 4: Repetition to be deleted-Affective attitude and burden

Good spot – now removed.
Page 15 Line no. 214 to 215: requires rewording for clarity. Cannot understand the statement made. No. of sites will be reduced to maintain treatment fidelity?

Original statement:

Treatment fidelity is particularly important for multi-site studies to understand if the intervention is operationalised in the same way across sites and minimise site by treatment interactions.

Reworded statement: In multi-site studies, it is particularly important to assess treatment fidelity because if treatment effects vary by site, we can observe whether this is related to variations in the delivery of treatment.

Page 17 Line 266: required additional information. If participant doesnt come for follow up intervention, what steps would be taken-could be described

This question relates more to the trial than the process evaluation and has been addressed in the trial protocol:

“Intervention adherence will be monitored weekly by the research team at SHU by reviewing SLI participants’ attendance at supervised exercise sessions, to identify those who have dipped below 75% attendance. CESs at each NH site will upload data relating to attendance and completion of aerobic and resistance exercise components of the SLI after each session using a REDCap web-based reporting software. Researchers will download, compile, and clean the data on a weekly basis. The database will track adherence relative to the number of prescribed sessions to date and produce “alerts” once a SLI participant drops below 75% adherence. This alert with suggested actions will be sent onto the NH fitness manager who will identify and deliver behavioural support in line with the COM-B [[30](https://trialsjournal.biomedcentral.com/articles/10.1186/s13063-024-07989-y#ref-CR30)] model of behaviour change (i.e. data verification, identify required behavioural support and deliver behavioural support). If a participant’s attendance remains below 75%, a maximum two further alerts are sent. A record of all alerts sent and subsequent actions will be maintained.”

McNaught, E., Reale, S., Bourke, L. *et al.* *S*upported exercise *T*r*A*ining for *M*en w*I*th prostate ca*N*cer on *A*ndrogen deprivation therapy (STAMINA): study protocol for a randomised controlled trial of the clinical and cost-effectiveness of the STAMINA lifestyle intervention compared with optimised usual care, including internal pilot and parallel process evaluation. *Trials* **25**, 257 (2024). <https://doi.org/10.1186/s13063-024-07989-y>

Reviewer #3: The manuscript outlines a protocol for a process evaluation embedded within a lifestyle intervention trial for prostate cancer patients undergoing androgen deprivation therapy (ADT). This evaluation covers recruitment, fidelity, intervention reach, and organizational aspects. Below are some points for clarification and consideration to enhance the transparency and robustness of the evaluation.

Major Comments:

Validity of the Analysis for Endpoints Based on Questionnaires:
The primary endpoints, quality of life and fatigue, rely on self-reported questionnaires (FACT-Prostate and FACIT-Fatigue), which, while validated, may be subject to response bias and measurement variability. How will the study account for the inherent subjectivity and potential variability in self-reported data? Have any sensitivity analyses been considered to assess the robustness of findings against these potential biases?

This comment is relevant to the design of the trial endpoints but not the objectives of the process evaluation discussed herein.

Timing and Schedule of Endpoint Collection:
The manuscript mentions that process data will be collected at various points during and after the intervention, but it is not entirely clear how this aligns with the timing of the main trial endpoints (e.g., quality of life measured at 12 months). Could the authors clarify the alignment of process data collection with primary trial outcomes? Additionally, given the 12-month follow-up, how will potential attrition bias be managed, particularly in relation to the consistency of data collected from self-reported questionnaires over time?

Again, this comment is relevant to the design of the trial endpoints but not the objectives of the process evaluation discussed herein.

Fidelity and Intervention Delivery:
The manuscript highlights fidelity assessment as a key component of the process evaluation, utilizing audio recordings, direct observations, and checklists. Could the authors elaborate on how fidelity will be quantified and reported? Will there be a specific scoring system to assess adherence to intervention components? Additionally, how will deviations from the intervention protocol be addressed within the analysis framework?
Specific information about how fidelity is being assessed and scored is described in details in the fidelity analysis section – lines 292-296. Deviations from the intervention protocol are a matter for the trial analysis and not the process evaluation and will be described in detail in the statistical analysis plan which is awaiting publication. Where patterns of poor treatment fidelity occur, they will be reported as part of this process evaluation but not necessarily intervened upon.

Bias in Self-Reported Data:
While the process evaluation is comprehensive, it does not address potential bias in the subjective, self-reported data (e.g., physical activity and quality of life outcomes) and qualitative interviews. How will such bias be accounted for in the study, and what impact might this have on the interpretation of the study’s conclusions?

Again, this comment is relevant to the design of the trial endpoints but not the objectives of the process evaluation discussed herein.

Minor Comments:

Understanding Trial Recruitment Performance:
The process evaluation likely involves tracking recruitment metrics, assessing participant demographics, and gathering qualitative feedback from recruitment staff. Will the evaluation also examine factors that may hinder recruitment, such as through feedback from recruitment staff and potential participants who declined to participate?

As stated in Table 1 (58) and Table 3 interview data from healthcare professionals will explore topics such as this. In Table 1 (4) we report that we will collect data about reasons for declining to take part from participants.

Describing Intervention Reach, Dose Delivered, and Dose Received:
Intervention reach, dose delivered, and dose received will be assessed through metrics such as session attendance and participant engagement. Is there a plan to supplement these metrics with qualitative feedback from participants, such as through exit interviews or surveys, to provide more context on adherence challenges?

Yes, this is clearly described in the interview sampling section: we specifically target individuals with different levels of intervention adherence as part of our interview sampling framework to address this.
Exploring Organizational Implications for Embedding and Sustaining the Intervention:
Semi-structured interviews with HCPs, guided by the Normalization Process Theory (NPT), are intended to assess the feasibility of embedding the intervention within the NHS. Have the authors considered using focus groups alongside individual interviews to capture a broader range of organizational dynamics and collaborative elements?

These were conducted at earlier phases of intervention development see papers:

<https://journals.plos.org/plosone/article?id=10.1371/journal.pone.0197606>

<https://link.springer.com/article/10.1186/s12913-021-06266-x>
