# Supplementary material for: Process evaluation for the STAMINA randomised controlled trial: A protocol
Source: PLoS One. 2025 Jul 14;20(7):e0323275. doi: 10.1371/journal.pone.0323275 (PMC12258564; doi:10.1371/journal.pone.0323275)
Supplement: Attachment — Submitted filename: Plos response 2.docx [file pone.0323275.s006.docx]

PE protocol peer reviews response – round 2

# My response to editors

Reviewer #2: It makes sense, from your explanation, to use continuous numbers in table 1, from 1 to 59. So please keep it and not change it to A1, B2, etc which would be more complex.
However, one clarification is requested:
1. "Page 7 Line no. 150 and 19 and line number -Implementation team doing the process evaluation-Need to incorporate suggestion to strengthen the manuscript-Are these going to be evaluated pre and post intervention?
*Though it is unclear what is being asked here, please refer to Fig 2 to see the timelines for evaluation of the process evaluation components in relation to intervention delivery."*
To clarify my query-This is regarding using implementation team for process evaluation. Though this is not ideal, funding limitation is an understandable reason for this compromise, which has already been mentioned as a limitation. What my suggestion regarding this is to add few precautions/strategies that would be undertaken to reduce bias in process evaluation reporting.

Thank you for the clarification. We believe the main strategy that we are able to employ to reduce reporting biases is to publish this protocol as now described on Pg 27, L313-317.

“….which has the potential to create biases in the collection and reporting of process evaluation data. In publishing this protocol, we intend to mitigate against the impact of these potential biases by acknowledging their potential in the data collection process for and by making explicit what data is to be collected, how each data type will be used to describe specific process evaluation components and how the process evaluation components and frameworks relate to each other.”

Publishing process evaluation protocols is still a rare practice with no guidelines though it is becoming more common.

Other methods to reduce reporting biases include inclusion of the interview topic guides in the Supplementary materials. We have also already stated that “At least 20% of data will be independently double or triple coded” with regard to interview analysis. We also have made clear where our findings will be triangulated using data from multiple sources (table 1).

Reviewer #3: Thank you for addressing all the comments.

The responses adequately address the reviewer’s concerns and provide relevant updates to the manuscript. The authors demonstrate an awareness of the study’s exploratory nature and the inherent limitations, which is appropriate for the study design.
